# The evaluation and application of multilocus variable number tandem repeat analysis (MLVA) for the molecular epidemiological study of *Salmonella enterica* subsp. *enterica* serovar Enteritidis infection

**DOI:** 10.1186/s12941-016-0119-3

**Published:** 2016-01-29

**Authors:** Yao Liu, Xiaolu Shi, Yinghui Li, Qiongcheng Chen, Min Jiang, Wanli Li, Yaqun Qiu, Yiman Lin, Yixiang Jiang, Biao Kan, Qun Sun, Qinghua Hu

**Affiliations:** Key Laboratory of Bio-resources and Eco-environment of the Ministry of Education, College of Life Sciences, Sichuan University, 29# Wangjiang Road, Chengdu, 610064 Sichuan People’s Republic of China; Shenzhen Major Infectious Disease Control Key Laboratory, Shenzhen Center for Disease Control and Prevention, Shenzhen, 518055 Guangdong People’s Republic of China; School of Life Sciences, Shenzhen University, Shenzhen, 518000 Guangdong People’s Republic of China; Key Laboratory of Surveillance and Early-warning on Infectious Disease, Division of Infectious Disease, Chinese Center for Disease Control and Prevention, Beijing, People’s Republic of China

**Keywords:** *S.* Enteritidis, MLVA, PFGE, Epidemiological study

## Abstract

**Background:**

*Salmonella enterica* subsp. *enterica* serovar Enteritidis (*S.* Enteritidis) is one of the most prevalent *Salmonella* serotypes that cause gastroenteritis worldwide and the most prevalent serotype causing *Salmonella* infections in China. A rapid molecular typing method with high throughput and good epidemiological discrimination is urgently needed for detecting the outbreaks and finding the source for effective control of *S*. Enteritidis infections.

**Methods:**

In this study, 194 strains which included 47 from six outbreaks that were well-characterized epidemiologically were analyzed with pulse field gel electrophoresis (PFGE) and multilocus variable number tandem repeat analysis (MLVA). Seven VNTR loci published by the US Center for Disease Control and Prevention (CDC) were used to evaluate and develop MLVA scheme for *S.* Enteritidis molecular subtyping by comparing with PFGE, and then MLVA was applied to the suspected outbreaks detection. All *S.* Enteritidis isolates were analyzed with MLVA to establish a MLVA database in Shenzhen, Guangdong province, China to facilitate the detection of *S*. Enteritidis infection clusters.

**Results:**

There were 33 MLVA types and 29 PFGE patterns among 147 sporadic isolates. These two measures had Simpson indices of 0.7701 and 0.8043, respectively, which did not differ significantly. Epidemiological concordance was evaluated by typing 47 isolates from six epidemiologically well-characterized outbreaks and it did not differ for PFGE and MLVA. We applied the well established MLVA method to detect two *S.* Enteritidis foodborne outbreaks and find their sources successfully in 2014. A MLVA database of 491 *S.* Enteritidis strains isolated from 2004 to 2014 was established for the surveillance of clusters in the future.

**Conclusions:**

MLVA typing of *S.* Enteritidis would be an effective tool for early warning and epidemiological surveillance of *S.* Enteritidis infections.

**Electronic supplementary material:**

The online version of this article (doi:10.1186/s12941-016-0119-3) contains supplementary material, which is available to authorized users.

## Background

The bacterium *Salmonella* causes acute gastroenteritis and it is one of the most common and widely distributed foodborne pathogens. There are two species of *Salmonella*: *Salmonellabongori* and *Salmonellaenterica*. Over 2500 different serotypes of *Salmonella enterica* species have been identified to date [1]. The global burden of non-typhoidal *Salmonella* gastroenteritis is heavy—there are 93.8 million cases annually leading to 155,000 deaths, of which 80.3 million cases are foodborne [2]. Despite the great number of serotypes, the most common foodborne pathogens in human *Salmonella* infections are *S.* Enteritidis and *S.* Typhimurium, which account for 57 % of all isolates from human cases, while *S.* Enteritidis is the most prevalent serotype in China [3].

Molecular typing methods for discriminating different bacterial isolates of the same species are essential epidemiological tools for detecting the outbreak of foodborne diseases [4]. An approprite molecular typing method must provide both strong discriminatory power and high epidemiological concordance. Discriminatory power is the probability that two unrelated isolates can be distinguished from one another, while epidemiological concordance is the probability to determine whether two outbreaks are due to a common source and whether two isolates are part of the same outbreak. When a typing method can offer these two critical abilities, additional characteristics including rapidity, high throughput, and good reproducibility are expected. The molecular typing methods that are used commonly to detect bacterial disease outbreaks include PFGE, MLVA, and clustered regularly interspaced short palindromic repeat and multiple-virulence-locus sequence typing (CRISPR-MVLST). Recently, powered by whole-genome-sequencing (WGS) technologies, whole-genome single-nucleotide polymorphism (SNP) typing (WGST) has been applied to resolve outbreak clusters of *S.* Enteritidis [5].

PulseNet [6], a national molecular subtyping network for the surveillance of foodborne disease, was established by the US CDC in 1996. It uses PFGE to identify clusters of patients infected with enteric bacteria that have indistinguishable PFGE patterns and hence possibly represent a common source of outbreaks. The initial PFGE method was used to type a single pathogen (*Escherichia coli* O157:H7) [7], and then further developed to enable typing of various clinically important bacteria, including *Listeria monocytogenes, Vibrio parahaemolyticus* and *Salmonella*. Combined with epidemiological investigations, PFGE has been used as an elementary typing tool for the analysis of transmission events of foodborne pathogens and used successfully in detection of outbreaks and effective control of the infections [8–11]. Although PFGE is currently considered as the gold standard for subtyping *Salmonella*, it is labor-intensive, time-consuming, and offers low throughput. Furthermore the standardized PFGE protocols require a trained and skilled technician and its implementation takes approximately three days.

MLVA is a molecular subtyping method based on amplification and fragment size analysis of tandem sequence repeats that are found in microbial genome of most bacterial species. It utilizes the naturally occurring variation in the number of tandem repeat DNA sequences. MLVA is rapid and highly reproducible, and the results can be easy to be interpreted and standardized among laboratories. Bacteria that have been typed by MLVA include *S.* Typhimurium [12, 13], *S.* Enteritidis [14, 15], *E. coli* O157:H7 [16, 17], and *Vibrio parahaemolyticus* [18]. In recent years, MLVA has been proposed as a supplement to PFGE for subtyping *S.* Enteritidis in many countries. It can connect suspected, fast-evolving bacterial strains to an outbreak even when they might look the same when analyzed by PFGE. In Europe, a standardized protocol is being used for *S.* Enteritidis based on a scheme with five VNTR loci [14, 19, 20]. In contrast, the standardized protocol for *S.* Enteritidis in the US uses seven VNTR loci [21]. The two standardized protocols share five VNTR loci.

A latest study compared four subtyping methods including WGST, CRISPR-MVLST, MLVA and PFGE to analyze outbreak clusters of *S.* Enteritidis [5]. MLVA outperformed CRISPR-MVLST and PFGE in delineating outbreak clusters. WGST provides superior discriminatory power and accurate phylogenetic inferences with high epidemiological correlation; however this technology relies on detailed bioinformatics analysis. Among the four subtyping methods, WGST was the most expensive and not feasible to be a routine subtyping method.

In Guangdong province in China, the *S.* Enteritidis serotype is the second most common cause of human salmonellosis [22]. Data from the laboratory-based diarrheal disease sentinel surveillance network collected in Shenzhen, Guangdong province, China since 2007 show that the peak positive rate of *Salmonella* infection increased from 3.82 % in 2007 to 14.36 % in 2014, and that *S.* Enteritidis was the second most prevalent serotype causing *Salmonella* infection in Shenzhen (Shenzhen CDC, unpublished data). Considering the rapidity, high throughput, moderate discriminatory ability and simple operation, MLVA holds the potential to be suitable for detecting the clusters of foodborne pathogens in provincial and local labs. The feasibility of using MLVA typing method for the epidemiological study and the detecting of local outbreaks has not yet been evaluated systematically in China so far.

In our study, we described the development, evaluation, and application of MLVA. Furthermore, we applied MLVA successfully to issue reports on *S.* Enteritidis infection outbreaks in 2014 and find their sources, and also established a MLVA database in Shenzhen for the surveillance of *S.* Enteritidis clusters. The results demonstrated that MLVA could be used to improve the efficiency of subtyping and the effectiveness of epidemiologically investigating *S.* Enteritidis infections.

## Methods

### Bacterial strains and DNA extraction

Bacterial strains used in this study were obtained from Shenzhen CDC in China between 2004 and 2014. The laboratory-based diarrheal disease sentinel surveillance network that established by the Shenzhen CDC collected patients information, including demographic characteristics (name, sex, age, nation and so on), recent travel, food consumption prior to becoming sick and clinical characteristics (date of accident, sampling date, isolation date and so on). Considering *S.* Enteritidis isolates’ epidemiological information, a total of 147 sporadic isolates (Additional file 1: Table S1) were selected for evaluating seven VNTR loci, developing MLVA and comparing discriminatory power between PFGE and MLVA. The 47 strains (Additional file 2: Table S2) from six epidemiologically well-characterized outbreaks (outbreaks 1, 2, 3, 4, 5 and 6; involving 6, 20, 4, 4, 8, and 5 isolates, respectively) were used to evaluate epidemiological concordance. All isolates were stored at −80 °C in 25 % glycerol, until they were allowed to grow overnight in 2 mL Luria–Bertani (LB) broth in a shaking incubator at 37 °C. Suspensions of bacterial cells were boiled for 5 min and used directly in the PCR reactions after a brief centrifugation at 12,000 rpm for 5 min.

### Measurement of systematic error in the genetic analysis

We used the CEQ 8800 Genetic Analysis System (Beckman Coulter Inc., Fullerton, CA, USA) to analyze the product size resulting from the amplification of VNTR loci. Sequences of all seven VNTR loci primers and characteristics are shown in Additional file 3: Table S3. To measure systematic error for the seven loci that we used, 31 *S.* Enteritidis strains were selected for PCR amplification and DNA sequencing. The following formula was used in MLVA to calculate the actual size of the repeat:

$${\text{Size}}\,{\text{of}}\,{\text{the}}\,{\text{repeat}} = {{\left[ {\left( {{\text{A}} \pm {\text{B}}} \right) - {\text{C}}} \right]} \mathord{\left/ {\vphantom {{\left[ {\left( {{\text{A}} \pm {\text{B}}} \right) - {\text{C}}} \right]} {\text{D}}}} \right. \kern-0pt} {\text{D}}}$$

where A is observed PCR product size, B is the systematic error, C is the flank that does not contain repeats, and D is the length of one repeat unit.

### Modification of the MLVA method

The amplification of SE6 described by the US CDC [21] was weak in our laboratory, so we modified the multiplex amplifications. Briefly, one multiplex amplification including SE1, SE2, SE6, and SE8 was carried out in a 20 μL volume containing 1 μL of template DNA, 2 μL of 10× PCR buffer, 1.6 μL of 2.5 mM dNTPs, 1.6 μL of 25 mM MgCl_2_, and 10 mM primer pairs: 0.08 μL SE1, 0.2 μL SE2, 0.6 μL SE6, and 0.25 μL SE8. Another multiplex amplification including SE3, SE5 and SE9 was carried out in a 20 μL volume containing 1 μL of template DNA, 2 μL of 10× PCR buffer, 1.6 μL of 2.5 mM dNTPs, 1.2 μL of 25 mM MgCl_2_, and 10 mM primer pairs: 0.3 μL SE3, 0.15 μL SE5, and 0.05 μL SE9. “S13130” was a clinical strain that presented in 31 sequenced isolates and used as a positive control in MLVA. Reaction mixtures without the DNA template were used as negative controls. After multiplex PCR, each multiplex reaction product was diluted 1:50 with PCR grade water. The next steps were performed according to the standardized protocols used by the US CDC on a Beckman Coulter CEQ 8800 GeXP for PCR fragment length analysis. The resulting data were imported into BioNumerics v5.1 software (Applied Maths) for clustering analysis, using categorical coefficients of zero tolerance and the unweighted pair-group method with arithmetic mean (UPGMA).

### PFGE

Sample preparation, restriction digestion, electrophoresis, and gel staining for PFGE followed the standardized protocols [23]. To provide a universal size standard, DNA from *Salmonella* Braenderup H9812 was restricted with XbaI [24]. All *S.* Enteritidis isolates were digested with the restriction endonuclease XbaI and the results were analyzed by BioNumerics v5.1 software (Applied Maths) then submitted to PulseNet China. PFGE patterns were compared on dendrograms generated in BioNumerics using the Dice coefficient and a 1.5 % band position tolerance. Patterns with no noticeable differences were considered indistinguishable and were assigned the same PFGE pattern.

### Statistical analysis

Diversity of each VNTR locus was assessed using Nei’s measure of allelic diversity [25]. Simpson’s diversity and Shannon-Weiner (Shannon) diversity indices were calculated to compare the discriminatory power of PFGE and MLVA. Variances and confidence intervals (CIs) of Simpson indices were calculated as described previously [26], and a *t* test was used to compare indices for MLVA versus PFGE.

## Results

### VNTR analysis

Diversity indices were calculated for individual locus based on 147 sporadic *S.* Enteritidis isolates, and the values ranged from 0.027 for SE8–0.722 for SE5 (Table 1). Among the seven VNTR loci, four of them (SE1, SE2, SE5 and SE9) showed appropriate discriminating abilities that were similar to previous reports [27–29]. In contrast, SE3, SE6 and SE8, were less polymorphic, with indices less than 0.1.

**Table 1:** Genetic diversity of seven VNTR loci in sporadic *S.* Enteritidis isolates

Locus	Repeat size (bp)	No. of variants^a^	Nei’s diversity index^b^
SE1	7	10	0.294
SE2	7	11	0.274
SE3	12	3	0.040
SE5	6	10	0.722
SE6	33	4	0.053
SE8	87	2	0.027
SE9	9	2	0.245

^a^number of different fragment size polymorphisms detected among 147 sporadic *S.* Enteritidis isolates

^b^Nei’s diversity index as 1 − Σ (allele frequency)^2^

### Diversity of MLVA and PFGE among the sporadic isolates

There were 33 MLVA types and 29 PFGE patterns among the sporadic isolates (Table 2), with a Simpson index of 0.7701 and 0.8043, respectively. Both the Simpson’s and Shannon’s indices were higher for PFGE than for MLVA, but there was no statistical difference between the two methods in terms of either index (*t* tests, *P* > 0.05). In contrast, most previous reports except one [30] found that the MLVA typing method showed much higher discriminatory power. There were common types for each method. JEGX01.CN0001 and JEGX01.CN0003 were common PFGE patterns in Shenzhen city and were also common in PulseNet China [31], while JEGMT0002 and JEGMT0003 were common MLVA types in Shenzhen.

**Table 2:** Comparison of MLVA and PFGE in sporadic *S.* Enteritidis isolates (n = 147)

Method	No. of types	Common types (n)	Diversity index^a^	
			Simpson’s (CI)	Shannon’s^b^
PFGE	29	JEGX01.CN0001 (33) JEGX01.CN0003 (55)	0.8043 (0.7536–0.8550)	2.2647
MLVA	33	JEGMT0002 (61) JEGMT0003 (33)	0.7701 (0.7146–0.8257)	2.1313

^a^ *P* values were > 0.05 for PFGE vs MLVA comparison of both diversity indices

^b^Shannon’s index is an indicator of species richness

### Epidemiological concordance of MLVA among isolates from well-characterized outbreaks

**Fig. 1:**
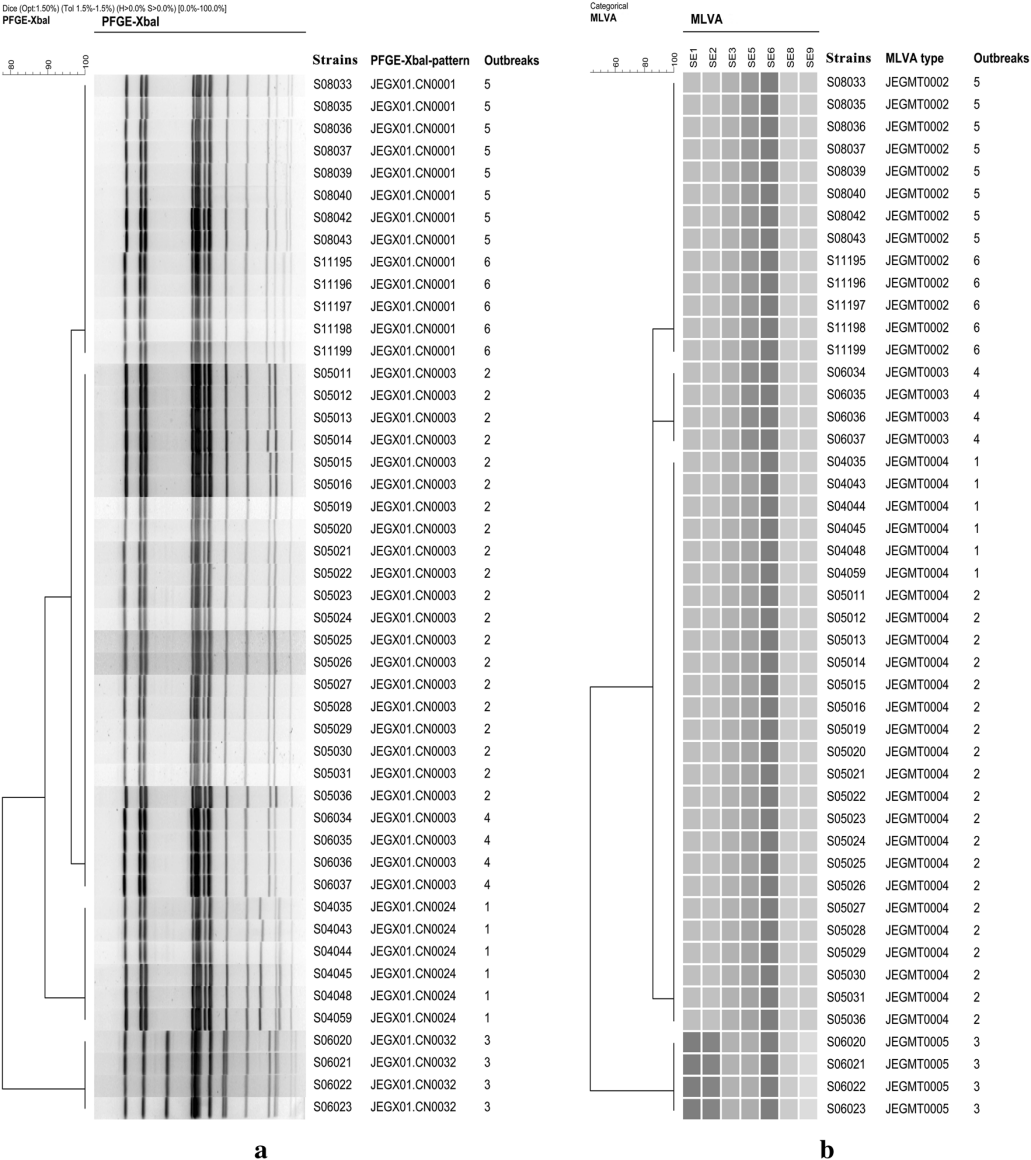
Dendrograms for PFGE **a** using restriction enzyme XbaI and MLVA **b** performed on six *S.* Enteritidis outbreaks. The PFGE dendrogram was generated using the Dice coefficient (optimization 1.5 % and position tolerance 1.5 %) and unweighted-pair group method with arithmetic averages algorithm (UPGMA) algorithm. The MLVA dendrogram was generated using the categorical coefficient and UPGMA algorithm

The 47 strains from six epidemiologically well-characterized outbreaks were divided into four MLVA types and four PFGE patterns. All isolates within a single outbreak were indistinguishable by both methods (Fig. 1). JEGX01.CN0001 and JEGX01.CN0003 were common PFGE patterns in outbreak isolates and were also common among the sporadic isolates. JEGMT0002 and JEGMT0004 were common MLVA types in the isolates from outbreaks, and strains linked to outbreak four, five and six had the common MLVA types among the sporadic isolates.

### Applying MLVA to detect *S.* Enteritidis infection outbreaks

**Fig. 2:**
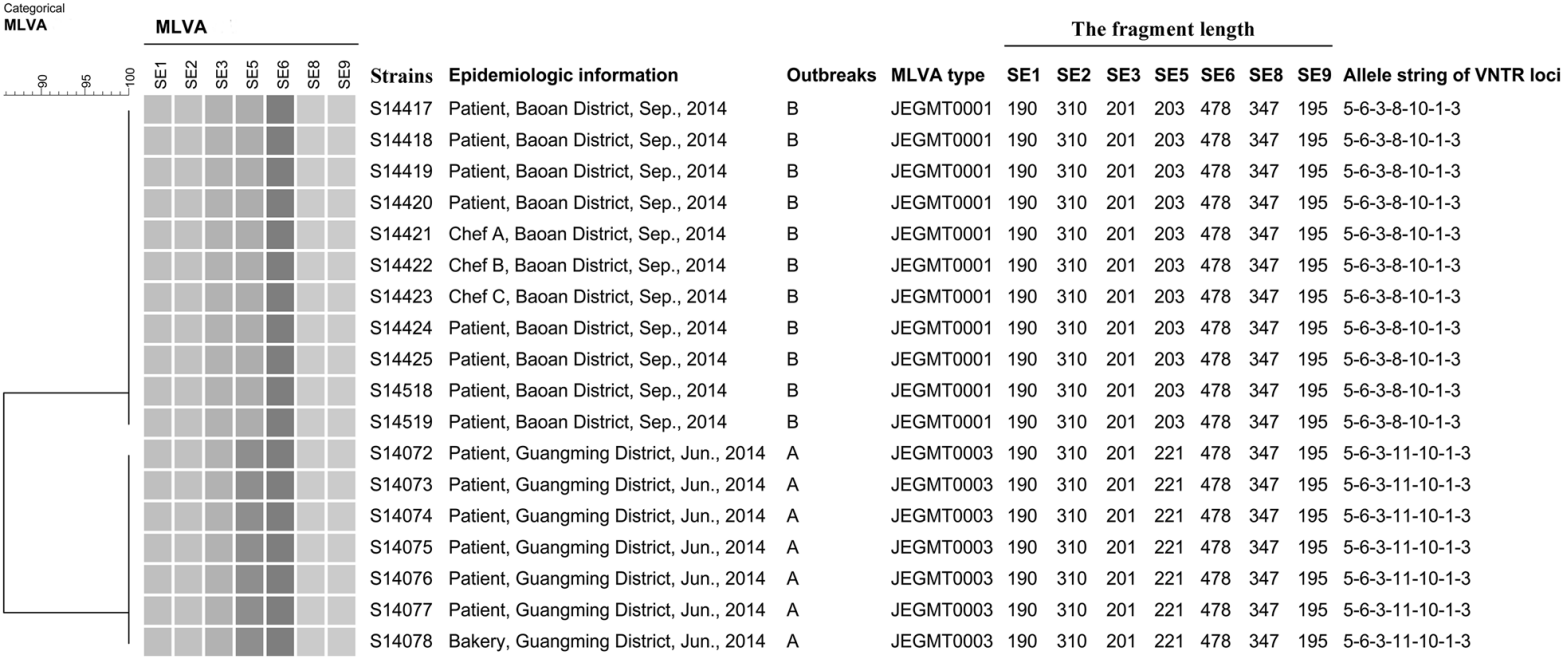
Dendrogram of 18 isolates evaluated by MLVA on two *S*. Enteritidis outbreaks in 2014. The MLVA dendrogram was generated using the categorical coefficient and unweighted pair group method with arithmetic means. “Allele string of VNTR loci” in the figure means a string of the actual number of repeats at each locus (in order of SE1–SE2–SE3–SE5–SE6–SE8–SE9)

We applied MLVA to detect two *S.* Enteritidis infection outbreaks in 2014 and find their sources successfully (Fig. 2).

In June 2014, a cluster of seven *S.* Enteritidis isolates with the common MLVA type JEGMT0003 was detected (Fig. 2). Epidemiological investigations showed that it was an outbreak of *S.* Enteritidis infection with the six strains being epidemiologically related and we confirmed that a bread countertop prepared with contaminated cakes was the food vehicle. As a result, the infectious source was decontaminated.

In September 2014, the Xixiang People’s Hospital reported two food-related outbreaks: two different groups of people (3 and 5 individuals, respectively) became sick after eating lunch in the same restaurant within one week. From patients’ stool samples, we isolated and identified the pathogen as *S.* Enteritidis, and used MLVA to type the isolates. A cluster of eight *S.* Enteritidis isolates with the same uncommon MLVA type, JEGMT0001, was detected (Fig. 2). The epidemiologists from Shenzhen CDC did the field investigation to confirm whether two outbreaks were epidemiologically related and where the sources were from. Based on the epidemiological investigation, 49 samples including 14 from foods, 10 environment swabs from kitchen and 25 anal swabs from 25 staffs who worked in the restaurant were collected and were sent to our laboratory for isolation and MLVA typing. As a result, three strains from anal swabs of three chefs shared the same JEGMT0001 MLVA type. Results of the epidemiological investigation and MLVA showed that two outbreaks were epidemiologically related and shared the same source.

### Establishment of a MLVA database

**Fig. 3:**
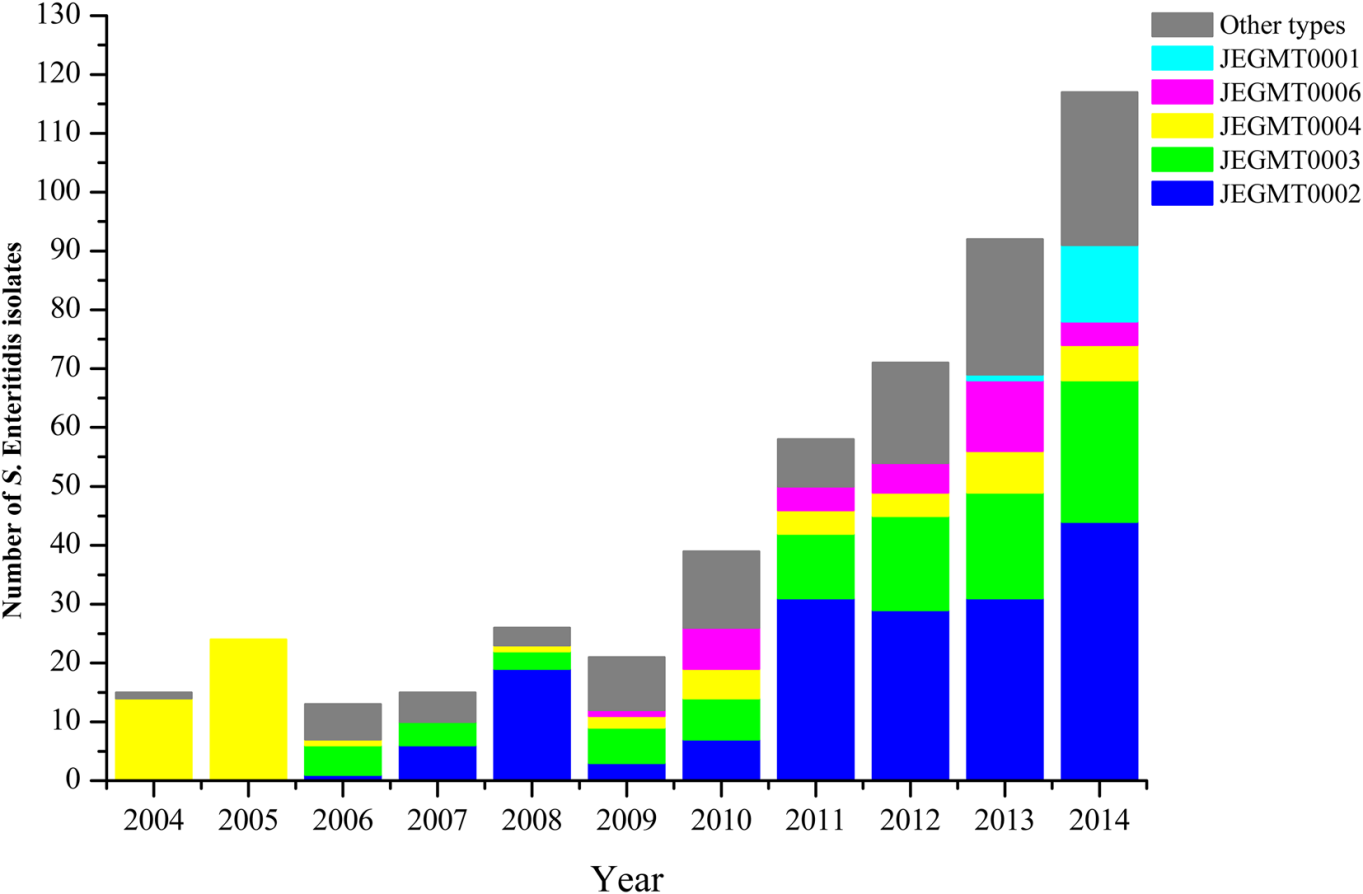
Annual distribution of Shenzhen *S.* Enteritidis isolates over an 11-year period (2004–2014) according to the MLVA database. Other types: all MLVA types with less than 2.8 % occurrence in the database. The number of JEGMT0001 type was 14 in the MLVA database and the proportion of the type was 2.8 %, so we defined other types as all MLVA types with less than 2.8 % occurrence in the MLVA database

We established a MLVA database of 491 strains that were isolated from 2004 to 2014 for the surveillance of *S.* Enteritidis clusters (Fig. 3). There were 70 MLVA types among all *S.* Enteritidis isolates, with JEGMT0002, JEGMT0003 and JEGMT0004 being the top three most common (Fig. 3). Although JEGMT0004 was a common type in the database, it was most common in 2004 and 2005 and the proportion of this type decreased in 2006.

## Discussion

Shenzhen is a coastal region of China that has expanded meteorically, from a village of 30,000 people in 1978 to a large city of more than 10 million people in 2010 [32]. The floating population is larger than the registered permanent population of this city. Data from the laboratory-based diarrheal disease sentinel surveillance network in Shenzhen showed that the rate of *S.* Enteritidis infection increased annually (the peak positive rate of *Salmonella* infection increased from 3.82 % in 2007 to 14.36 % in 2014; Shenzhen CDC, unpublished data) and that *S.* Enteritidis infection was the second most prevalent serotype causing *Salmonella* infections. It is essential to type pathogens as soon as possible, in order to detect suspected infection outbreaks and to find the source in this kind of situation. The standardized PFGE protocol for subtyping *Salmonella* takes approximately three days; in contrast, the MLVA typing can be finished within 6 h. Although PFGE is widely considered to be the gold standard for subtyping *Salmonella*, implementation of this typing method for massive strains is not feasible. Hence, evaluating and developing MLVA for *S.* Enteritidis is very important for detecting outbreaks and tracing their sources.

The Simpson’s and Shannon’s diversity indices were higher for PFGE than for MLVA. However, the MLVA typing method showed much higher discriminatory power than PFGE in studies from other countries [5, 15, 27, 28]. Although the two indices were higher for PFGE than for MLVA, the values were not significantly different. To raise discriminatory power of MLVA, four more loci (SENTR2, SENTR3, SE-7, SE-10) described by previous studies [27, 29] were added into MLVA, but it did not result in a further discrimination (data not shown). Compared with discriminatory power, clustering concordance with epidemiological data is an equally important measure for evaluation of the utility of the molecular subtyping method. Epidemiological concordance did not differ for PFGE and MLVA in our assessment.

We succeeded in applying the MLVA typing to detect two *S.* Enteritidis foodborne outbreaks in 2014 and find their sources, and a MLVA database of all *S.* Enteritidis isolated from the years 2004 to 2014 was established for the surveillance of *S.* Enteritidis clusters. These results indicated that MLVA would be a useful tool for early warning and epidemiological surveillance of *S.* Enteritidis infection. In the latest study, whole-genome WGST showed the highest discriminatory power of four subtyping methods (WGST, CRISPR-MVLST, MLVA and PFGE), and the discriminatory power between CRISPR-MVLST and MLVA was similar [5]. We used CRISPR-MVLST to analyze three outbreaks (data not shown), and the typing result of CRISPR-MVLST was the same as MLVA. Two outbreaks shared the same CRISPR-MVLST type, while they shared the same MLVA type. Although epidemiological concordance between CRISPR-MVLST and MLVA was same, the expense of CRISPR-MVLST was tripled compared with MLVA. MLVA may be the most time-saving and the least expensive method for subtyping *S.* Enteritidis.

Whether PFGE or MLVA typing was used, there were common types for each method. It seems that regardless of the primary molecular subtyping method, a secondary subtyping method would be needed to differentiate the common *S.* Enteritidis subtypes. Hence, MLVA has been proposed as a supplementary method to PFGE for subtyping *S.* Enteritidis in previous studies. In July 2010, the US CDC calculated the average of JEGX01.0004 that was the most common PFGE pattern in the PulseNet USA database and identified a nationwide sustained increase in the number of the type, and the epidemiological investigation then showed that it was a nationwide *S.* Enteritidis infection outbreak associated with shell eggs [33]. In this study, we propose MLVA as an alternative to PFGE for subtyping *S.* Enteritidis. Typically, an epidemiological investigation would be initiated when the MLVA type of three or more strains within 30 days is shown to be identical. However, JEGMT0002 and JEGMT0003 were common MLVA types in Shenzhen, so that practice might increase the investigation workload of the epidemiologists. The average of JEGMT0002 and JEGMT0003 based on our MLVA database can be calculated, and average of the two types is 5 and 4 respectively. When cases of common MLVA types are larger than the average within 30 days, an epidemiological investigation would then be initiated to confirm whether or not it is an outbreak. An epidemiological investigation is needed when three or more clinical strains with same uncommon MLVA types or new types that do not appear previously are detected within 30 days. JEGMT0001 was an uncommon MLVA type that appeared in 2013 along with one clinical isolate and the type of 13 strains were detected in 2014, and then the epidemiological investigation confirmed that it was the outbreak.

Although all of the *S.* Enteritidis isolates evaluated in this study were from Shenzhen city, the common PFGE patterns of isolates were representative of the most common PFGE patterns in PulseNet China [31]. Nevertheless, in this study there was a limitation in the number of illness cases. Still, further investigation is necessary to estimate the average of two common MLVA types for detecting the clusters.

## Conclusion

In conclusion, MLVA for *S.* Enteritidis showed a satisfactory performance, in terms of simplicity, speed, and reproducibility. The method can be applied as a routine subtyping method for early warning of outbreaks and initiating epidemiological investigations.

## Additional files

10.1186/s12941-016-0119-3 Clinical isolates from sporadic cases used in this study.
10.1186/s12941-016-0119-3 Forty-seven isolates from six epidemiologically well-characterized outbreaks in this study.
10.1186/s12941-016-0119-3 Primer sequences and PCR product size used for *S.* Enteritidis MLVA typing.

## Abbreviations

*S.* Enteritidis: *Salmonella enterica* subsp. *enterica* serovar Enteritidis
*S.* Typhimurium: *Salmonella enterica* subsp. *enterica* serovar Typhimurium
VNTR: variable number tandem repeat
MLVA: multilocus variable number tandem repeat analysis
PFGE: pulse field gel electrophoresis
PCR: polymerase chain reaction
CDC: Center for Disease Control and Prevention
CRISPR-MVLST: clustered regularly interspaced short palindromic repeat and multiple-virulence-locus sequence typing
WGS: whole-genome-sequencing
SNP: single-nucleotide polymorphism
WGST: whole-genome single-nucleotide polymorphism typing
LB: Luria–Bertani
