# Supplementary material for: The evaluation and application of multilocus variable number tandem repeat analysis (MLVA) for the molecular epidemiological study of Salmonella enterica subsp. enterica serovar Enteritidis infection
Source: Ann Clin Microbiol Antimicrob. 2016 Jan 29;15:4. doi: 10.1186/s12941-016-0119-3 (PMC4731957; doi:10.1186/s12941-016-0119-3)
Supplement: Supplementary file 1 — 10.1186/s12941-016-0119-3 Clinical isolates from sporadic cases used in this study. [file 12941_2016_119_MOESM1_ESM.doc]

**Supplementary Table 1. Clinical isolates from sporadic cases used in this study**

| Isolates | MLVA type | PFGE pattern ^a^ | Date of accident | Location details | Allele string of VNTR loci ^b^ |
| --- | --- | --- | --- | --- | --- |
| S04346 | JEGMT0004 | JEGX01.CN0024 | 2004/12/2 | Shajing People’s Hospital | 5-6-3-9-10-1-3 |
| S05093 | JEGMT0004 | JEGX01.CN0003 | 2005/7/14 | Futian CDC | 5-6-3-9-10-1-3 |
| S06025 | JEGMT0018 | JEGX01.CN0338 | 2006/7/6 | Shenzhen Second People's Hospital | 6-5-3-12-14-1-2 |
| S06032 | JEGMT0003 | JEGX01.CN0001 | 2006/8/7 | Futian CDC | 5-6-3-11-10-1-3 |
| S07016 | JEGMT0002 | JEGX01.CN0001 | 2007/9/3 | Longhua People’s Hospital | 5-6-3-10-10-1-3 |
| S07019 | JEGMT0003 | JEGX01.CN0224 | 2007/8/17 | Longgang District Central Hospital | 5-6-3-11-10-1-3 |
| S07022 | JEGMT0002 | JEGX01.CN0001 | 2007/9/30 | Nanshan CDC | 5-6-3-10-10-1-3 |
| S07027 | JEGMT0022 | JEGX01.CN0078 | 2007/10/12 | Xixiang People’s Hospital | 5-10-3-7-8-1-2 |
| S07033 | JEGMT0002 | JEGX01.CN0001 | 2007/11/9 | Pinghu People’s Hospital | 5-6-3-10-10-1-3 |
| S08003 | JEGMT0003 | JEGX01.CN0003 | 2008/2/27 | Luohu People’s Hospital | 5-6-3-11-10-1-3 |
| S08004 | JEGMT0002 | JEGX01.CN0003 | 2008/3/13 | Shenzhen CDC | 5-6-3-10-10-1-3 |
| S08006 | JEGMT0002 | JEGX01.CN0001 | 2008/3/18 | Xixiang People’s Hospital | 5-6-3-10-10-1-3 |
| S08014 | JEGMT0002 | JEGX01.CN0001 | 2008/4/10 | Peking University Shenzhen Hospital | 5-6-3-10-10-1-3 |
| S08046 | JEGMT0003 | JEGX01.CN0003 | 2008/6/29 | Longhua Preventive Health Care | 5-6-3-11-10-1-3 |
| S08094 | JEGMT0002 | JEGX01.CN0003 | 2008/8/11 | Peking University Shenzhen Hospital | 5-6-3-10-10-1-3 |
| S08099 | JEGMT0004 | JEGX01.CN0003 | 2008/8/12 | Xixiang People’s Hospital | 5-6-3-9-10-1-3 |
| S08105 | JEGMT0002 | JEGX01.CN0001 | 2008/8/25 | Xili People’s Hospital | 5-6-3-10-10-1-3 |
| S08122 | JEGMT0002 | JEGX01.CN0003 | 2008/9/18 | Peking University Shenzhen Hospital | 5-6-3-10-10-1-3 |
| S08128 | JEGMT0009 | JEGX01.CN0003 | 2008/12/8 | Shajing People’s Hospital | 6-6-3-11-10-1-3 |
| S09006 | JEGMT0006 | JEGX01.CN0003 | 2009/3/25 | Xixiang People’s Hospital | 5-6-3-12-10-1-3 |
| S09018 | JEGMT0015 | JEGX01.CN0184 | 2009/4/27 | Peking University Shenzhen Hospital | 6-13-3-7-10-1-2 |
| S09026 | JEGMT0047 | JEGX01.CN0184 | 2009/6/9 | Buji People’s Hospital | 6-11-3-7-10-1-2 |
| S09032 | JEGMT0036 | JEGX01.CN0339 | 2009/6/7 | Shenzhen People’s Hospital | 3-6-3-13-10-1-2 |
| S09035 | JEGMT0003 | JEGX01.CN0001 | 2009/5/27 | Luohu People’s Hospital | 5-6-3-11-10-1-3 |
| Isolates | MLVA type | PFGE pattern ^a^ | Date of accident | Location details | Allele string of VNTR loci ^b^ |
| S09049 | JEGMT0004 | JEGX01.CN0019 | 2009/7/17 | Longhua People’s Hospital | 5-6-3-9-10-1-3 |
| S09059 | JEGMT0003 | JEGX01.CN0003 | 2009/8/10 | Baoan CDC | 5-6-3-11-10-1-3 |
| S09076 | JEGMT0002 | JEGX01.CN0001 | 2009/10/19 | Nanshan CDC | 5-6-3-10-10-1-3 |
| S09078 | JEGMT0003 | JEGX01.CN0001 | 2009/9/2 | Longgang District Central Hospital | 5-6-3-11-10-1-3 |
| S09081 | JEGMT0017 | JEGX01.CN0332 | 2009/10/11 | Baoan CDC | 6-9-3-9-10-1-2 |
| S09088 | JEGMT0003 | JEGX01.CN0016 | 2009/11/14 | Xixiang People’s Hospital | 5-6-3-11-10-1-3 |
| S09122 | JEGMT0004 | JEGX01.CN0001 | 2009/12/3 | Shajing People’s Hospital | 5-6-3-9-10-1-3 |
| S09131 | JEGMT0002 | JEGX01.CN0003 | 2009/12/22 | Shenzhen People’s Hospital | 5-6-3-10-10-1-3 |
| S09133 | JEGMT0003 | JEGX01.CN0016 | 2009/12/23 | Buji People’s Hospital | 5-6-3-11-10-1-3 |
| S10013 | JEGMT0006 | JEGX01.CN0003 | 2010/4/16 | Peking University Shenzhen Hospital | 5-6-3-12-10-1-3 |
| S10015 | JEGMT0006 | JEGX01.CN0050 | 2010/4/6 | Longgang People's Hospital | 5-6-3-12-10-1-3 |
| S10020 | JEGMT0002 | JEGX01.CN0001 | 2010/6/4 | Xixiang People’s Hospital | 5-6-3-10-10-1-3 |
| S10023 | JEGMT0004 | JEGX01.CN0003 | 2010/6/12 | Longgang District Central Hospital | 5-6-3-9-10-1-3 |
| S10033 | JEGMT0006 | JEGX01.CN0050 | 2010/6/18 | Gongming People's Hospital | 5-6-3-12-10-1-3 |
| S10036 | JEGMT0003 | JEGX01.CN0003 | 2010/6/3 | Longhua People’s Hospital | 5-6-3-11-10-1-3 |
| S10037 | JEGMT0008 | JEGX01.CN0003 | 2010/6/30 | Shekou People's Hospital | 5-7-3-10-10-1-3 |
| S10043 | JEGMT0003 | JEGX01.CN0001 | 2010/7/6 | Peking University Shenzhen Hospital | 5-6-3-11-10-1-3 |
| S10046 | JEGMT0033 | JEGX01.CN0003 | 2010/7/13 | Xixiang People’s Hospital | 6-6-3-12-10-1-3 |
| S10063 | JEGMT0002 | JEGX01.CN0003 | 2010/7/9 | Shekou People's Hospital | 5-6-3-10-10-1-3 |
| S10092 | JEGMT0039 | JEGX01.CN0001 | 2010/10/19 | Longgang District Central Hospital | 5-6-3-10-14-1-3 |
| S10103 | JEGMT0002 | JEGX01.CN0001 | 2010/10/21 | Gongming People's Hospital | 5-6-3-10-10-1-3 |
| S10135 | JEGMT0002 | JEGX01.CN0001 | 2010/11/24 | Shenzhen Second People's Hospital | 5-6-3-10-10-1-3 |
| S10143 | JEGMT0004 | JEGX01.CN0003 | 2010/11/13 | Longhua People’s Hospital | 5-6-3-9-10-1-3 |
| S10144 | JEGMT0011 | JEGX01.CN0078 | 2010/12/10 | Shenzhen Fourth People's Hospital | 7-9-3-7-10-1-2 |
| S10150 | JEGMT0012 | JEGX01.CN0032 | 2010/9/27 | Buji People’s Hospital | 10-9-3-7-10-1-2 |
| Isolates | MLVA type | PFGE pattern ^a^ | Date of accident | Location details | Allele string of VNTR loci ^b^ |
| S10152 | JEGMT0020 | JEGX01.CN0335 | 2010/12/30 | Luohu People’s Hospital | 3-5-3-23-10-1-2 |
| S11012 | JEGMT0003 | JEGX01.CN0003 | 2011/5/11 | Longgang People's Hospital | 5-6-3-11-10-1-3 |
| S11016 | JEGMT0002 | JEGX01.CN0001 | 2011/5/5 | Shajing People’s Hospital | 5-6-3-10-10-1-3 |
| S11049 | JEGMT0002 | JEGX01.CN0001 | 2011/6/30 | Nanshan People's Hospital | 5-6-3-10-10-1-3 |
| S11054 | JEGMT0002 | JEGX01.CN0340 | 2011/6/2 | Longgang District Central Hospital | 5-6-3-10-10-1-3 |
| S11059 | JEGMT0003 | JEGX01.CN0016 | 2011/7/22 | Shenzhen People’s Hospital | 5-6-3-11-10-1-3 |
| S11061 | JEGMT0006 | JEGX01.CN0003 | 2011/7/27 | Shenzhen Children's Hospital | 5-6-3-12-10-1-3 |
| S11065 | JEGMT0002 | JEGX01.CN0092 | 2011/7/20 | Shajing People’s Hospital | 5-6-3-10-10-1-3 |
| S11073 | JEGMT0003 | JEGX01.CN0003 | 2011/8/10 | Longgang District Central Hospital | 5-6-3-11-10-1-3 |
| S11097 | JEGMT0004 | JEGX01.CN0003 | 2011/8/3 | Nanshan People's Hospital | 5-6-3-9-10-1-3 |
| S11114 | JEGMT0003 | JEGX01.CN0003 | 2011/9/7 | Longgang District Central Hospital | 5-6-3-11-10-1-3 |
| S11116 | JEGMT0002 | JEGX01.CN0001 | 2011/9/7 | Gongming People's Hospital | 5-6-3-10-10-1-3 |
| S11134 | JEGMT0006 | JEGX01.CN0003 | 2011/8/31 | Shenzhen Children's Hospital | 5-6-3-12-10-1-3 |
| S11145 | JEGMT0002 | JEGX01.CN0092 | 2011/9/22 | Futian CDC | 5-6-3-10-10-1-3 |
| S11147 | JEGMT0002 | JEGX01.CN0003 | 2011/9/28 | Longgang People's Hospital | 5-6-3-10-10-1-3 |
| S11149 | JEGMT0002 | JEGX01.CN0003 | 2011/10/11 | Gongming People's Hospital | 5-6-3-10-10-1-3 |
| S11179 | JEGMT0003 | JEGX01.CN0001 | 2011/11/3 | Nanshan People's Hospital | 5-6-3-11-10-1-3 |
| S11193 | JEGMT0035 | JEGX01.CN0078 | 2011/10/26 | Longgang People's Hospital | 6-10-3-7-10-1-2 |
| S11201 | JEGMT0002 | JEGX01.CN0001 | 2011/11/6 | Xili People’s Hospital | 5-6-3-10-10-1-3 |
| S11206 | JEGMT0002 | JEGX01.CN0001 | 2011/11/24 | Luohu Maternal and Child Health Hospital | 5-6-3-10-10-1-3 |
| S11250 | JEGMT0006 | JQPX01.CN0023 | 2011/12/28 | Shekou People's Hospital | 5-6-3-12-10-1-3 |
| S12005 | JEGMT0002 | JEGX01.CN0004 | 2012/1/28 | Nanshan People's Hospital | 5-6-3-10-10-1-3 |
| S12013 | JEGMT0023 | JEGX01.CN0003 | 2012/2/26 | Nanshan People's Hospital | 5-6-3-6-10-1-3 |
| S12020 | JEGMT0002 | JEGX01.CN0092 | 2012/5/12 | Xixiang People’s Hospital | 5-6-3-10-10-1-3 |
| S12023 | JEGMT0002 | JEGX01.CN0005 | 2012/3/14 | Longgang People's Hospital | 5-6-3-10-10-1-3 |
| Isolates | MLVA type | PFGE pattern ^a^ | Date of accident | Location details | Allele string of VNTR loci ^b^ |
| S12030 | JEGMT0002 | JEGX01.CN0003 | 2012/5/27 | Longhua People’s Hospital | 5-6-3-10-10-1-3 |
| S12039 | JEGMT0002 | JEGX01.CN0003 | 2012/6/20 | Xixiang People’s Hospital | 5-6-3-10-10-1-3 |
| S12042 | JEGMT0002 | JEGX01.CN0001 | 2012/6/28 | Longgang District Central Hospital | 5-6-3-10-10-1-3 |
| S12058 | JEGMT0002 | JEGX01.CN0005 | 2012/6/26 | Gongming People's Hospital | 5-6-3-10-10-1-3 |
| S12063 | JEGMT0003 | JEGX01.CN0016 | 2012/6/24 | Xixiang People’s Hospital | 5-6-3-11-10-1-3 |
| S12066 | JEGMT0026 | JEGX01.CN0032 | 2012/6/5 | Shajing People’s Hospital | 8-13-3-8-10-1-2 |
| S12072 | JEGMT0002 | JEGX01.CN0003 | 2012/7/2 | Futian CDC | 5-6-3-10-10-1-3 |
| S12085 | JEGMT0002 | JEGX01.CN0330 | 2012/7/12 | Shajing People’s Hospital | 5-6-3-10-10-1-3 |
| S12088 | JEGMT0003 | JEGX01.CN0003 | 2012/6/21 | Nanshan People's Hospital | 5-6-3-11-10-1-3 |
| S12091 | JEGMT0003 | JEGX01.CN0019 | 2012/7/20 | Xixiang People’s Hospital | 5-6-3-11-10-1-3 |
| S12097 | JEGMT0002 | JEGX01.CN0003 | 2012/7/29 | Longgang People's Hospital | 5-6-3-10-10-1-3 |
| S12100 | JEGMT0003 | JEGX01.CN0003 | 2012/7/25 | Shajing People’s Hospital | 5-6-3-11-10-1-3 |
| S12101 | JEGMT0002 | JEGX01.CN0092 | 2012/7/6 | Shenzhen Children's Hospital | 5-6-3-10-10-1-3 |
| S12108 | JEGMT0003 | JEGX01.CN0003 | 2012/6/30 | Longgang People's Hospital | 5-6-3-11-10-1-3 |
| S12116 | JEGMT0003 | JEGX01.CN0019 | 2012/7/7 | Gongming People's Hospital | 5-6-3-11-10-1-3 |
| S12121 | JEGMT0025 | JEGX01.CN0078 | 2012/8/16 | Peking University Shenzhen Hospital | 9-9-3-8-10-1-2 |
| S12127 | JEGMT0042 | JEGX01.CN0341 | 2012/7/29 | Nanshan People's Hospital | 0-0-0-14-11-1-2 |
| S12131 | JEGMT0004 | JEGX01.CN0189 | 2012/8/6 | Longgang People's Hospital | 5-6-3-9-10-1-3 |
| S12140 | JEGMT0041 | JEGX01.CN0332 | 2012/8/23 | Futian CDC | 4-8-4-10-10-2-2 |
| S12143 | JEGMT0007 | JEGX01.CN0016 | 2012/8/9 | Nanshan CDC | 5-6-3-13-10-1-3 |
| S12155 | JEGMT0029 | JEGX01.CN0032 | 2012/8/20 | Futian TCM Hospital | 11-15-3-9-10-1-2 |
| S12160 | JEGMT0009 | JEGX01.CN0003 | 2012/9/5 | Gongming People's Hospital | 6-6-3-11-10-1-3 |
| S12175 | JEGMT0002 | JEGX01.CN0005 | 2012/9/10 | Xixiang People’s Hospital | 5-6-3-10-10-1-3 |
| S12177 | JEGMT0002 | JEGX01.CN0200 | 2012/9/6 | Shajing People’s Hospital | 5-6-3-10-10-1-3 |
| S12181 | JEGMT0040 | JEGX01.CN0003 | 2012/8/5 | Longgang District Central Hospital | 5-7-3-11-10-1-3 |
| Isolates | MLVA type | PFGE pattern ^a^ | Date of accident | Location details | Allele string of VNTR loci ^b^ |
| S12182 | JEGMT0002 | JEGX01.CN0092 | 2012/9/7 | Gongming People's Hospital | 5-6-3-10-10-1-3 |
| S12208 | JEGMT0013 | JEGX01.CN0032 | 2012/10/5 | Shajing People’s Hospital | 7-18-3-7-10-1-2 |
| S12212 | JEGMT0002 | JEGX01.CN0005 | 2012/9/10 | Longgang District Central Hospital | 5-6-3-10-10-1-3 |
| S12243 | JEGMT0004 | JEGX01.CN0007 | 2012/9/21 | Peking University Shenzhen Hospital | 5-6-3-9-10-1-3 |
| S12249 | JEGMT0003 | JEGX01.CN0003 | 2012/11/16 | Futian CDC | 5-6-3-11-10-1-3 |
| S12253 | JEGMT0002 | JEGX01.CN0003 | 2012/11/2 | Shajing People’s Hospital | 5-6-3-10-10-1-3 |
| S12257 | JEGMT0002 | JEGX01.CN0342 | 2012/10/21 | Longgang District Central Hospital | 5-6-3-10-10-1-3 |
| S12258 | JEGMT0002 | JEGX01.CN0003 | 2012/11/18 | Nanshan People's Hospital | 5-6-3-10-10-1-3 |
| S12261 | JEGMT0002 | JEGX01.CN0003 | 2012/11/21 | Baoan Maternal and Child Health Hospital | 5-6-3-10-10-1-3 |
| S12264 | JEGMT0038 | JEGX01.CN0003 | 2012/10/29 | Peking University Shenzhen Hospital | 4-6-3-12-10-1-3 |
| S12270 | JEGMT0003 | JEGX01.CN0003 | 2012/11/26 | Longhua People’s Hospital | 5-6-3-11-10-1-3 |
| S13009 | JEGMT0003 | JEGX01.CN0016 | 2012/11/2 | Futian CDC | 5-6-3-11-10-1-3 |
| S13012 | JEGMT0002 | JEGX01.CN0003 | 2012/11/17 | Buji People’s Hospital | 5-6-3-10-10-1-3 |
| S13015 | JEGMT0003 | JEGX01.CN0003 | 2013/1/11 | Shajing People’s Hospital | 5-6-3-11-10-1-3 |
| S13035 | JEGMT0003 | JEGX01.CN0016 | 2013/3/28 | Baoan Maternal and Child Health Hospital | 5-6-3-11-10-1-3 |
| S13050 | JEGMT0004 | JEGX01.CN0001 | 2013/5/22 | Peking University Shenzhen Hospital | 5-6-3-9-10-1-3 |
| S13055 | JEGMT0035 | JEGX01.CN0074 | 2013/5/9 | Shajing People’s Hospital | 6-10-3-7-10-1-2 |
| S13056 | JEGMT0002 | JEGX01.CN0003 | 2013/5/1 | Xixiang People’s Hospital | 5-6-3-10-10-1-3 |
| S13061 | JEGMT0002 | JEGX01.CN0003 | 2013/5/1 | Nanshan People's Hospital | 5-6-3-10-10-1-3 |
| S13066 | JEGMT0016 | JEGX01.CN0032 | 2013/5/7 | Guangming CDC | 8-13-3-7-10-1-2 |
| S13077 | JEGMT0002 | JEGX01.CN0001 | 2013/5/21 | Longgang People's Hospital | 5-6-3-10-10-1-3 |
| S13080 | JEGMT0002 | JEGX01.CN0003 | 2013/7/2 | Shenzhen TCM Hospital | 5-6-3-10-10-1-3 |
| S13082 | JEGMT0002 | JEGX01.CN0001 | 2013/7/9 | Pingshan CDC | 5-6-3-10-10-1-3 |
| S13088 | JEGMT0002 | JEGX01.CN0176 | 2013/6/30 | Songgang People’s Hospital | 5-6-3-10-10-1-3 |
| S13099 | JEGMT0002 | JEGX01.CN0003 | 2013/4/25 | Longgang District Central Hospital | 5-6-3-10-10-1-3 |
| Isolates | MLVA type | PFGE pattern ^a^ | Date of accident | Location details | Allele string of VNTR loci ^b^ |
| S13114 | JEGMT0003 | JEGX01.CN0001 | 2013/6/22 | Xixiang People’s Hospital | 5-6-3-11-10-1-3 |
| S13129 | JEGMT0003 | JEGX01.CN0003 | 2013/7/6 | Songgang People’s Hospital | 5-6-3-11-10-1-3 |
| S13130 | JEGMT0002 | JEGX01.CN0003 | 2013/7/9 | Guanlan People’s Hospital | 5-6-3-10-10-1-3 |
| S13151 | JEGMT0002 | JEGX01.CN0001 | 2013/6/19 | Futian CDC | 5-6-3-10-10-1-3 |
| S13154 | JEGMT0004 | JEGX01.CN0003 | 2013/4/28 | Futian CDC | 5-6-3-9-10-1-3 |
| S13166 | JEGMT0003 | JEGX01.CN0001 | 2013/7/29 | Longgang CDC | 5-6-3-11-10-1-3 |
| S13184 | JEGMT0016 | JEGX01.CN0032 | 2013/8/16 | Guangming CDC | 8-13-3-7-10-1-2 |
| S13187 | JEGMT0003 | JEGX01.CN0001 | 2013/7/20 | Shajing People’s Hospital | 5-6-3-11-10-1-3 |
| S13198 | JEGMT0002 | JEGX01.CN0003 | 2013/8/28 | Songgang People’s Hospital | 5-6-3-10-10-1-3 |
| S13231 | JEGMT0004 | JEGX01.CN0001 | 2013/9/22 | Futian CDC | 5-6-3-9-10-1-3 |
| S13234 | JEGMT0031 | JEGX01.CN0194 | 2013/8/24 | Baoan CDC | 4-6-3-10-10-1-3 |
| S13248 | JEGMT0003 | JEGX01.CN0016 | 2013/8/21 | Longhua People’s Hospital | 5-6-3-11-10-1-3 |
| S13252 | JEGMT0032 | JQPX01.CN0023 | 2013/10/14 | Xixiang People’s Hospital | 5-7-3-14-10-1-3 |
| S13261 | JEGMT0002 | JEGX01.CN0003 | 2013/10/11 | Longgang People's Hospital | 5-6-3-10-10-1-3 |
| S13270 | JEGMT0002 | JEGX01.CN0005 | 2013/9/2 | Peking University Shenzhen Hospital | 5-6-3-10-10-1-3 |
| S13286 | JEGMT0002 | JEGX01.CN0003 | 2013/10/9 | Shajing People’s Hospital | 5-6-3-10-10-1-3 |
| S13294 | JEGMT0002 | JEGX01.CN0343 | 2013/11/10 | Futian CDC | 5-6-3-10-10-1-3 |
| S13327 | JEGMT0006 | JEGX01.CN0019 | 2013/11/26 | Longhua People’s Hospital | 5-6-3-12-10-1-3 |
| S13330 | JEGMT0002 | JEGX01.CN0003 | 2013/11/1 | Songgang People’s Hospital | 5-6-3-10-10-1-3 |
| S13339 | JEGMT0002 | JEGX01.CN0001 | 2013/9/17 | Nanshan People's Hospital | 5-6-3-10-10-1-3 |
| S13346 | JEGMT0010 | JEGX01.CN0332 | 2013/12/4 | Luohu People’s Hospital | 5-6-4-8-10-2-2 |
| S13361 | JEGMT0003 | JEGX01.CN0001 | 2013/11/10 | Guangming CDC | 5-6-3-11-10-1-3 |
| S14002 | JEGMT0019 | JEGX01.CN0032 | 2013/12/14 | Shajing People’s Hospital | 8-5-3-12-10-1-2 |

^a^ The PFGE type in PulseNet China that using restriction enzyme XbaI.

^b^ A string of the actual number of repeats at each locus, in order of SE1-SE2-SE3-SE5-SE6-SE8-SE9.
