# Supplementary material for: The evaluation and application of multilocus variable number tandem repeat analysis (MLVA) for the molecular epidemiological study of Salmonella enterica subsp. enterica serovar Enteritidis infection
Source: Ann Clin Microbiol Antimicrob. 2016 Jan 29;15:4. doi: 10.1186/s12941-016-0119-3 (PMC4731957; doi:10.1186/s12941-016-0119-3)
Supplement: Supplementary file 2 — 10.1186/s12941-016-0119-3 Forty-seven isolates from six epidemiologically well-characterized outbreaks in this study. [file 12941_2016_119_MOESM2_ESM.doc]

**Supplementary Table 2. 47 isolates from six epidemiologically well-characterized outbreaks in this study**

| Isolates | Outbreaks | Epidemiologic information | MLVA type | PFGE pattern ^a^ | Allele string of VNTR loci ^b^ |
| --- | --- | --- | --- | --- | --- |
| S04035 | 1 | Sandwich, Baoan District, Sep., 2004 | JEGMT0004 | JEGX01.CN0024 | 5-6-3-9-10-1-3 |
| S04043 | 1 | Patient, Baoan District, Sep., 2004 | JEGMT0004 | JEGX01.CN0024 | 5-6-3-9-10-1-3 |
| S04044 | 1 | Patient, Baoan District, Sep., 2004 | JEGMT0004 | JEGX01.CN0024 | 5-6-3-9-10-1-3 |
| S04045 | 1 | Patient, Baoan District, Sep., 2004 | JEGMT0004 | JEGX01.CN0024 | 5-6-3-9-10-1-3 |
| S04048 | 1 | Patient, Baoan District, Sep., 2004 | JEGMT0004 | JEGX01.CN0024 | 5-6-3-9-10-1-3 |
| S04059 | 1 | Patient, Baoan District, Sep., 2004 | JEGMT0004 | JEGX01.CN0024 | 5-6-3-9-10-1-3 |
| S05011 | 2 | Patient, Nanshan District, Jun., 2005 | JEGMT0004 | JEGX01.CN0003 | 5-6-3-9-10-1-3 |
| S05012 | 2 | Patient, Nanshan District, Jun., 2005 | JEGMT0004 | JEGX01.CN0003 | 5-6-3-9-10-1-3 |
| S05013 | 2 | Patient, Nanshan District, Jun., 2005 | JEGMT0004 | JEGX01.CN0003 | 5-6-3-9-10-1-3 |
| S05014 | 2 | Patient, Nanshan District, Jun., 2005 | JEGMT0004 | JEGX01.CN0003 | 5-6-3-9-10-1-3 |
| S05015 | 2 | Patient, Nanshan District, Jun., 2005 | JEGMT0004 | JEGX01.CN0003 | 5-6-3-9-10-1-3 |
| S05016 | 2 | Patient, Nanshan District, Jun., 2005 | JEGMT0004 | JEGX01.CN0003 | 5-6-3-9-10-1-3 |
| S05019 | 2 | Patient, Nanshan District, Jun., 2005 | JEGMT0004 | JEGX01.CN0003 | 5-6-3-9-10-1-3 |
| S05020 | 2 | Patient, Nanshan District, Jun., 2005 | JEGMT0004 | JEGX01.CN0003 | 5-6-3-9-10-1-3 |
| S05021 | 2 | Patient, Nanshan District, Jun., 2005 | JEGMT0004 | JEGX01.CN0003 | 5-6-3-9-10-1-3 |
| S05022 | 2 | Patient, Nanshan District, Jun., 2005 | JEGMT0004 | JEGX01.CN0003 | 5-6-3-9-10-1-3 |
| S05023 | 2 | Patient, Nanshan District, Jun., 2005 | JEGMT0004 | JEGX01.CN0003 | 5-6-3-9-10-1-3 |
| S05024 | 2 | Patient, Nanshan District, Jun., 2005 | JEGMT0004 | JEGX01.CN0003 | 5-6-3-9-10-1-3 |
| S05025 | 2 | Patient, Nanshan District, Jun., 2005 | JEGMT0004 | JEGX01.CN0003 | 5-6-3-9-10-1-3 |
| S05026 | 2 | Patient, Nanshan District, Jun., 2005 | JEGMT0004 | JEGX01.CN0003 | 5-6-3-9-10-1-3 |
| S05027 | 2 | Patient, Nanshan District, Jun., 2005 | JEGMT0004 | JEGX01.CN0003 | 5-6-3-9-10-1-3 |
| S05028 | 2 | Patient, Nanshan District, Jun., 2005 | JEGMT0004 | JEGX01.CN0003 | 5-6-3-9-10-1-3 |
| S05029 | 2 | Patient, Nanshan District, Jun., 2005 | JEGMT0004 | JEGX01.CN0003 | 5-6-3-9-10-1-3 |
| S05030 | 2 | Patient, Nanshan District, Jun., 2005 | JEGMT0004 | JEGX01.CN0003 | 5-6-3-9-10-1-3 |
| Isolates | Outbreaks | Epidemiologic information | MLVA type | PFGE pattern ^a^ | Allele string of VNTR loci ^b^ |
| S05031 | 2 | Patient, Nanshan District, Jun., 2005 | JEGMT0004 | JEGX01.CN0003 | 5-6-3-9-10-1-3 |
| S05036 | 2 | Sandwich, Nanshan District, Jun., 2005 | JEGMT0004 | JEGX01.CN0003 | 5-6-3-9-10-1-3 |
| S06020 | 3 | Patient, Futian District, Jun., 2006 | JEGMT0005 | JEGX01.CN0032 | 10-12-3-8-10-1-2 |
| S06021 | 3 | Patient, Futian District, Jun., 2006 | JEGMT0005 | JEGX01.CN0032 | 10-12-3-8-10-1-2 |
| S06022 | 3 | Patient, Futian District, Jun., 2006 | JEGMT0005 | JEGX01.CN0032 | 10-12-3-8-10-1-2 |
| S06023 | 3 | Goose, Futian District, Jun., 2006 | JEGMT0005 | JEGX01.CN0032 | 10-12-3-8-10-1-2 |
| S06034 | 4 | Patient, Baoan District, Aug., 2006 | JEGMT0003 | JEGX01.CN0003 | 5-6-3-11-10-1-3 |
| S06035 | 4 | Patient, Baoan District, Aug., 2006 | JEGMT0003 | JEGX01.CN0003 | 5-6-3-11-10-1-3 |
| S06036 | 4 | Patient, Baoan District, Aug., 2006 | JEGMT0003 | JEGX01.CN0003 | 5-6-3-11-10-1-3 |
| S06037 | 4 | Restaurant, Baoan District, Aug., 2006 | JEGMT0003 | JEGX01.CN0003 | 5-6-3-11-10-1-3 |
| S08033 | 5 | Patient, Downtown Shenzhen, Jun., 2008 | JEGMT0002 | JEGX01.CN0001 | 5-6-3-10-10-1-3 |
| S08035 | 5 | Patient, Downtown Shenzhen, Jun., 2008 | JEGMT0002 | JEGX01.CN0001 | 5-6-3-10-10-1-3 |
| S08036 | 5 | Patient, Downtown Shenzhen, Jun., 2008 | JEGMT0002 | JEGX01.CN0001 | 5-6-3-10-10-1-3 |
| S08037 | 5 | Patient, Downtown Shenzhen, Jun., 2008 | JEGMT0002 | JEGX01.CN0001 | 5-6-3-10-10-1-3 |
| S08039 | 5 | Chicken, Downtown Shenzhen, Jun., 2008 | JEGMT0002 | JEGX01.CN0001 | 5-6-3-10-10-1-3 |
| S08040 | 5 | Patient, Downtown Shenzhen, Jun., 2008 | JEGMT0002 | JEGX01.CN0001 | 5-6-3-10-10-1-3 |
| S08042 | 5 | Patient, Downtown Shenzhen, Jun., 2008 | JEGMT0002 | JEGX01.CN0001 | 5-6-3-10-10-1-3 |
| S08043 | 5 | Patient, Downtown Shenzhen, Jun., 2008 | JEGMT0002 | JEGX01.CN0001 | 5-6-3-10-10-1-3 |
| S11195 | 6 | Patient, Nanshan District, Nov., 2011 | JEGMT0002 | JEGX01.CN0001 | 5-6-3-10-10-1-3 |
| S11196 | 6 | Patient, Nanshan District, Nov., 2011 | JEGMT0002 | JEGX01.CN0001 | 5-6-3-10-10-1-3 |
| S11197 | 6 | Patient, Nanshan District, Nov., 2011 | JEGMT0002 | JEGX01.CN0001 | 5-6-3-10-10-1-3 |
| S11198 | 6 | Patient, Nanshan District, Nov., 2011 | JEGMT0002 | JEGX01.CN0001 | 5-6-3-10-10-1-3 |
| S11199 | 6 | Restaurant, Nanshan District, Nov., 2011 | JEGMT0002 | JEGX01.CN0001 | 5-6-3-10-10-1-3 |

^a^ The PFGE type in PulseNet China that using restriction enzyme XbaI.

^b^ A string of the actual number of repeats at each locus, in order of SE1-SE2-SE3-SE5-SE6-SE8-SE9.
