# Supplementary material for: The evaluation and application of multilocus variable number tandem repeat analysis (MLVA) for the molecular epidemiological study of Salmonella enterica subsp. enterica serovar Enteritidis infection
Source: Ann Clin Microbiol Antimicrob. 2016 Jan 29;15:4. doi: 10.1186/s12941-016-0119-3 (PMC4731957; doi:10.1186/s12941-016-0119-3)
Supplement: Supplementary file 3 — 10.1186/s12941-016-0119-3 Primer sequences and PCR product size used for S. Enteritidis MLVA typing. [file 12941_2016_119_MOESM3_ESM.doc]

**Supplementary Table 3. Primer sequences and PCR product size used for *S.* Enteritidis MLVA typing**

| Locus | Primer sequence | Annealing temp (°C) | DNA sequence length in bp ^a^ (size CEQ 8800 Genetic Analysis System ^b^ ) |
| --- | --- | --- | --- |
| SE1 | F: (Cy5)TGTGGGACTGCTTCAACCTTTGGGC  R: CCAGCCATCCATACCAAGACCAACACTCTATGA | 65 | 185 (190) |
| SE2 | F: (Alexa fluor 750)GTGCTTCCTCAGGTTGCTTTTAGCCTTGTTCG  R: GGGGAATGGACGGAGGCGATAGACG | 65 | 314 (310) |
| SE3 | F: (Alexa fluor 750)CGGGATAAGTGCCACATAACACAGTCGCTAAGC  R: CGCCAGTGTTAAAGGAATGAATGAACCTGCTGATG | 65 | 198 (201) |
| SE5 | F: (Cy5.5)GGCTGGCGGGAAACCACCATC  R: GCCGAACAGCAGGATCTGTCCATTAGTCACTG | 65 | 215 (215) |
| SE6 | F: (Cy5)CTGGTCGCAGGTGTGGC  R: GGTGACGCCGTTGCTGAAGGTAATAACAGAGTC | 65 | 477 (478) |
| SE8 | F: (Cy5.5)GGTAGCTTGCCGCATAGCAGCAGAAGT  R: GGCGGCAAGCGAGCGAATCC | 65 | 346 (347) |
| SE9 | F: (Cy5)CCACCTCTTTACGGATACTGTCCACCAGC  R: GGCGTTACTGGCGGCGTTCG | 65 | 191 (195) |

^a^ based on *S.* Enteritidis “S13130”, which was a clinical strain that presented in 31 sequenced isolates and used as a positive control in MLVA.

^b^ size analyzed using CEQ 8800 Genetic Analysis System (Beckman Coulter Inc., Fullerton, CA, USA).
